# konnect2prot: a web application to explore the protein properties in a functional protein–protein interaction network

**DOI:** 10.1093/bioinformatics/btac815

**Published:** 2022-12-21

**Authors:** Shivam Kumar, Dipanka Tanu Sarmah, Shailendra Asthana, Samrat Chatterjee

**Affiliations:** Complex Analysis Group, Translational Health Science and Technology Institute, NCR Biotech Science Cluster, Faridabad 121001, India; Complex Analysis Group, Translational Health Science and Technology Institute, NCR Biotech Science Cluster, Faridabad 121001, India; Non-communicable Disease Group, Translational Health Science and Technology Institute, NCR Biotech Science Cluster, Faridabad 121001, India; Complex Analysis Group, Translational Health Science and Technology Institute, NCR Biotech Science Cluster, Faridabad 121001, India

## Abstract

**Motivation:**

The regulation of proteins governs the biological processes and functions and, therefore, the organisms’ phenotype. So there is an unmet need for a systematic tool for identifying the proteins that play a crucial role in information processing in a protein–protein interaction (PPI) network. However, the current protein databases and web servers still lag behind to provide an end-to-end pipeline that can leverage the topological understanding of a context-specific PPI network to identify the influential spreaders. Addressing this, we developed a web application, ‘konnect2prot’ (k2p), which can generate context-specific directional PPI network from the input proteins and detect their biological and topological importance in the network.

**Results:**

We pooled together a large amount of ontological knowledge, parsed it down into a functional network, and gained insight into the molecular underpinnings of the disease development by creating a one-stop junction for PPI data. k2p contains both local and global information about a protein, such as protein class, disease mutations, ligands and PDB structure, enriched processes and pathways, multi-disease interactome and hubs and bottlenecks in the directional network. It also identifies spreaders in the network and maps them to disease hallmarks to determine whether they can affect the disease state or not.

**Availability and implementation:**

konnect2prot is freely accessible using the link https://konnect2prot.thsti.in. The code repository is https://github.com/samrat-lab/k2p_bioinfo-2022.

## 1 Introduction

Investigating the relatedness of proteins and thereby elucidating the mechanism of disease progression is a difficult undertaking. This daunting nature can be attributed to the lack of a concept that explores a synergistic association between the local and global properties of proteins. The former includes the knowledge of the available structures and ligands, mutations in various diseases or conditions, the class of the protein etc. The mode of protein–protein interactions (PPIs) - activations/inhibitions, also comes under the grasp of local properties. These properties play a crucial role in the landscape of drug discovery. Proteases, kinases, G protein-coupled receptors and nuclear hormone receptors, e.g. are the most often targeted proteins for which effective medicines have been produced (Bakheet and Doig, 2009). Information about ligands is also beneficial in the same context, as, in comparison to other ligands, soluble protein ligands, such as cytokines and growth hormones can be conveniently targeted with mAbs (Attwood *et al.*, 2020). Ligands help identify the protein’s binding sites, inhibitory and modulatory mechanisms and generate the basis for discovering novel drugs for the protein. The structure-guided knowledge and strategies are expected to promote the discovery of novel therapeutics targeting the orthosteric or allosteric sites, thereby improving the possibility of effectively and specifically treating and preventing the diseases (Mittal *et al.*, 2021a,b). The global properties, on the other hand, cover the aspect of enriched pathways, processes, molecular functions, multi-disease landscape etc. These properties are vital to identify pathways, processes, or functions that are mostly affected during a progression of a disease. Another crucial aspect is the topological insight of the PPI network, which sheds light on the key proteins governing the disease interactome (Anand *et al.*, 2018; Sarmah *et al.*, 2021b).

Distinguishing these key proteins that drive the disease progression has proven to be a daunting task, further exacerbated by the intricacy of understanding how such drivers interact synergistically. In the conventional approach, defining such drivers relies only on the topology of the PPI networks and not on their context-specificity. Methods akin to this have the drawback that only the topological properties of PPI networks alone do not capture the whole landscape of the signalling complexity. Therefore, the derived driver proteins may not be sufficient to illuminate the complexity of the mechanism of disease progression. Identifying a target necessitates the causal inferences about interacting partners, which must be augmented in specific contexts with the knowledge about pathways, localizations, diseases and biological processes.

There are various ways to identify these driver proteins. However, irrespective of the method, the identified proteins must exert a global effect on the network. Preferably, such proteins should not be derived using a degree-biased process. One of the suitable properties to measure this effect is spreading, which evaluates how influential a node is at disseminating information throughout a network. Spreading is a key activity in a network, and hence an unmet need exists for identifying the influential spreaders (IS) in a PPI network. It has been reported that the drug target proteins are generally better spreaders of disease (Perez-Lopez *et al.*, 2015). For best outcomes, these spreaders should be derived from a functional inference of interacting proteins, which, again, must be enriched in specific contexts by augmenting information, such as pathways, localization, process and tissue specificity. Proteins have evolved to perform specific functions within particular subcellular compartments. Based on cellular localization, the interactors of a protein and its effect on a cellular process may differ (Sarmah *et al.*, 2021a). Comprehending how proteins are localized in different subcellular compartments is an integral part of understanding the organization and function of the cell as a whole (Dönnes and Höglund, 2004). Again, the proteins present in various diseases help to pinpoint the multifaceted landscape of disease (Hammoud and Kramer, 2020). Information on pathways helps distinguish proteins that have a mutual impact on disease progression (Reimand *et al.*, 2019). Therefore, it is crucial to identify the influential pathways in the PPI network. Similarly, the case exists with biological processes and molecular functions (Tomczak *et al.*, 2018). They both refer to the organized actions undertaken by cells for the organism to function properly. The analysis of subnetworks, created upon the filtration of influential pathways, processes and functions, would provide more insight than the whole network as such filtrations identify relevant regions of a more extensive PPI network that may lead to the progression of a disease. Another crucial attribute of a PPI network is directionality (Anand *et al.*, 2018; Vinayagam *et al.*, 2016). The directionality in a PPI network refers to the functional relationship in the network, which captures the regulatory effect exerted by the source protein on the target protein. The identification of key proteins in an undirected network may lead to various false-positive results (Yu *et al.*, 2016). For instance, when the mode of interactions for drug–disease relationships is absent, we cannot determine if a drug heals a disease or produces one as a side effect (Yu *et al.*, 2015). Again, every disease is driven by or characterized by some hallmarks. The possibility of a protein being a potential target increases if it is associated with those hallmarks. Moreover, the relationships between protein targets and signalling pathway activity enable the development of a personalized treatment strategy, such as determining how to influence a particular pathway to maximize pharmaceutical response (Yang *et al.*, 2018).

**Fig. 1.**
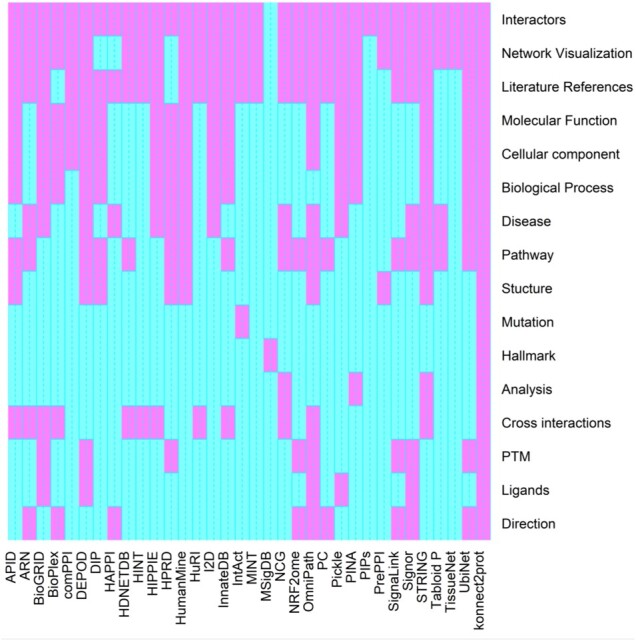
The attribute-database heat map. The absence or presence of an attribute in a database is represented by cyan, and violet colours, respectively. The figure shows that only k2p holds all the attributes compared to the other databases

Numerous databases containing the local and global information of proteins have been created to fulfil the need for the aggregation of PPI data for a more informed insight into the mechanisms of cells and diseases. Some of the popular PPI databases are BioGRID (Oughtred *et al.*, 2021), DIP (Xenarios *et al.*, 2000), MINT (Licata *et al.*, 2012), InnateDB (Breuer *et al.*, 2013), HPRD (Keshava Prasad *et al.*, 2009), comPPI (Veres *et al.*, 2015), STRING (Szklarczyk *et al.*, 2021), SIGNOR (Licata *et al.*, 2020), BioPlex (Huttlin *et al.*, 2015), IntAct (Hermjakob *et al.*, 2004), CellMap (Dallago *et al.*, 2017) etc. Though all of them provide information about interactors and interactions, cellular localization-based specific undirected interaction is only provided by cellmap and comPPI. Only STRING and InnateDB provide a tool for network analysis, that too in a limited context. Many databases like KEGG (Kanehisa and Goto, 2000), MINT, IntAct, DisGeNET (Piñero *et al.*, 2020) etc. have added ‘disease’ as an attribute to understand the association of proteins with diseases. To decipher the structural properties of proteins, various databases have been developed, including protein data bank (Berman *et al.*, 2000), PDBsum (Laskowski *et al.*, 2018) etc. Databases like HDMD (Stenson *et al.*, 2017), DoCM (Ainscough *et al.*, 2016) etc. store the information about disease mutations but do not provide other local or global properties of a protein. The comparison of attributes of various PPI databases is shown in Figure 1. All this information is required for a comprehensive understanding of a protein and its interaction. For seamless scientific investigations, such information needs to be curated, filtered and stored in one place. The creation of such a one-stop junction would help the researchers to gain comprehensive knowledge of human protein interactome and explore them based on their needs. However, the current PPI platforms still lag behind to provide an end-to-end pipeline that can leverage the topological understanding of a context-specific PPI network to identify the IS that can be further explored as disease targets.

**Fig. 2.**
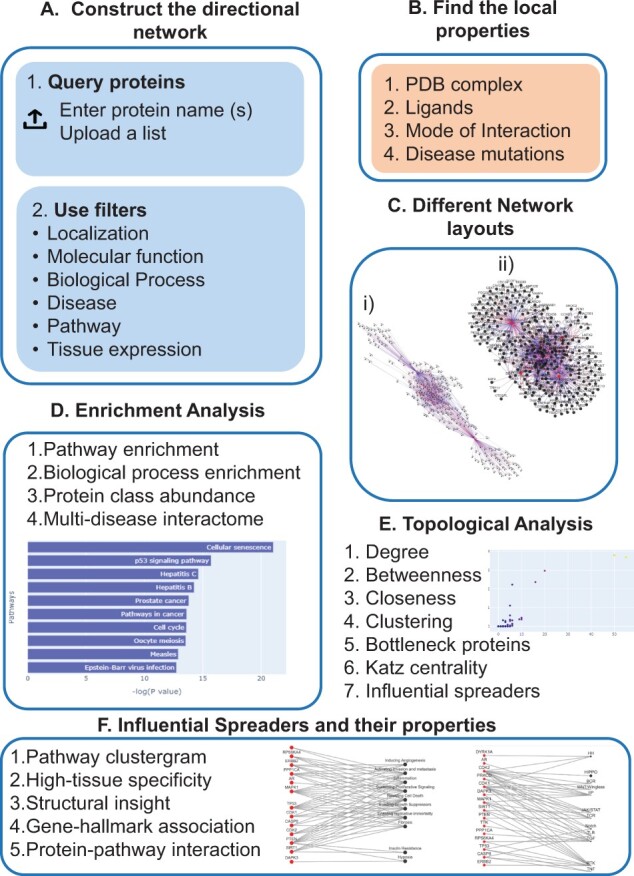
The overview of k2p. It allows users to identify the IS in a directional PPI network. (**A**) The directional network can be constructed by directly typing protein names or by uploading a list in the query box. Using the filters, the user can make the network context-specific. (**B**) The available local information of a protein. (**C**) The multiple network layouts available at k2p. (**D**) The four types of enrichment analysis used in k2p. (**E**) The topological analysis methods of the PPI network. The spreaders are identified using the VoteRank algorithm (Zhang *et al.*, 2016). (**F**) The various properties of the spreaders

With konnect2prot (k2p), we seek to fill this void and provide a simple stand-alone solution for constructing directional context-specific PPI networks based on pathways, subcellular localization, disease, molecular function and tissue specificity. It stores both local and global information about a protein, such as its class, disease mutations, ligands and PDB structure, enrichment processes, pathways, multi-disease interactome and identification of hubs and bottlenecks in its directional first neighbourhood PPI network. Additionally, k2p identifies the top IS within the constructed PPI network and then maps them to disease hallmarks to determine whether or not the identified spreaders are capable of affecting the disease state, a feat further bolstered by mapping the spreaders to crucial signalling pathways. k2p has been implemented as an easy-to-access web interface implemented in PYTHON libraries, and it can be accessed remotely in a user-friendly manner using contemporary web browsers and does not require the installation of additional plugins or applications. We present the overview of k2p in Figure 2.

## 2 Materials and methods

### 2.1 Data acquisition

The knowledge base of k2p is constructed by acquiring data from multiple databases. The SIGNOR (Licata *et al.*, 2020) and OmniPath (Türei *et al.*, 2021) databases were used to obtain the causal PPI interactions. The information on pathways was obtained from the KEGG (Kanehisa and Goto, 2000) and SignaLink (Csabai *et al.*, 2022). The disease-related information was taken from DisGeNET (Piñero *et al.*, 2020), and the information on ligands (which are the therapeutic modulators, such as small molecules, peptides etc.) and protein class was curated from DGIdb (Freshour *et al.*, 2021). Information on tissue expression and subcellular localization were taken from the Human Protein Atlas (Karlsson *et al.*, 2021). Biological process and molecular function information were curated from the GO database. The disease mutation and structural information were curated from DocM (Ainscough *et al.*, 2016) and PDB (Berman *et al.*, 2000), respectively. Finally, we performed an extensive literature survey to find the disease hallmarks and the related genes.

### 2.2 Analysis of the PPI network

#### 2.2. 1 Local information extraction

The local information in k2p can be subdivided into two parts. (i) Node-based information (clicking on a node in the network) – contains information on the PDB complex, ligands and disease mutation of a protein. (ii) Edge-based information (clicking on an edge in the network) – contains information on the interaction between two proteins and the mechanism and effect of the relationship. Each of these information panels again contains a cascade of information. For instance, the PDB complex palette contains information on the conducted experiment and resolution. All this information is extractable in .xlsx format.

#### 2.2. 2 Global information extraction

The global information of k2p spans through various pallets. They are: (i) functional enrichment: the gene ontology, pathway and disease enrichment are performed using Enrichr API (Chen *et al.*, 2013). The enrichment analysis provides a global landscape of the PPI network being investigated. For example, disease enrichment elucidates the multi-disease landscape, investigates biological topics in the context of disease and identifies previously unanticipated functionalities. (ii) Protein class abundance: the information on protein classes plays a vital role in the landscape of drug discovery. Taking this as motivation, k2p finds the class of proteins in the constructed directional PPI network and plots a bar graph of protein abundance in each class. This graph can be found in the protein class palate. (iii) Topological analysis of the network: the topological features of k2p include in- and out-degree centrality, betweenness centrality, clustering coefficient and closeness centrality. The topological comparison palette shows the degree versus betweenness plot to better understand the influence of hub proteins in the propagation of information throughout the network. (iv) Computational tools to predict the spreaders: to find the spreaders of the network, we have opted for the VoteRank algorithm (Zhang *et al.*, 2016). This algorithm tracks the IS in the network who have a high voting score as compared to their neighbour voting scores. At each iteration, the voting power of the elected spreader is zeroed while that of its neighbours is reduced by a factor. The top 15 proteins by VoteRank and their topological and local properties can be found in Section 3. All the images and tables obtained from the analysis are exportable. (v) Pathway clustergram of the spreaders: k2p generates a pathway clustergram to see the number of cross-talks between various pathways, which are influenced by the spreaders. This type of analysis uncovers the unique pathways of individual spreaders, which can be utilized to decipher disease-related changes. (vi) Tissue specificity of the spreaders: due to their increased safety, tissue-specific genes are regarded to be ideal therapeutic targets. The target’s tissue specificity may also be connected to the drug’s efficacy. The expression patterns of the spreaders can be obtained from the tissue-specificity panel to see whether, in the studied system, they are highly expressed or not. This information is crucial, because, for instance, if the studied system is non-alcoholic fatty liver disease (NAFLD), the potential target should be highly expressed in the liver. Otherwise, targeting it may increase the risk of adverse effects or unfavourable outcomes, which are not acceptable given the lengthy and costly process of drug development. (vii) Understanding the association of spreaders with diseases: we constructed a gene-hallmark directional bipartite network for a better understanding of the disease pathophysiology. A gene is mapped to a hallmark with evidence reported in the literature. Many spreaders may be implausible disease candidates, and such analysis can uncover false positives and filter proteins, resulting in a much smaller and more supported group of possible targets.

## 3 Results and discussion

### 3.1 Web interface

Users can start with single or multiple proteins as seeds with their gene symbols as inputs to explore the relationships between proteins. The first iteration displays the interactors of the seed proteins across all the biological processes, molecular functions, pathways, subcellular localization and various tissues. The network layout plays a critical role in unveiling important patterns during network visualization. We have provided various layouts, in the ‘layout’ option, like preset, random, grid, circle, concentric, breadth-first, the compound spring embedder (CoSE), CoSE-Bilkent, cola, Euler, spread, dagree and klay. The user can develop context-specific networks by using the filters in the left-hand panel.

**Fig. 3.**
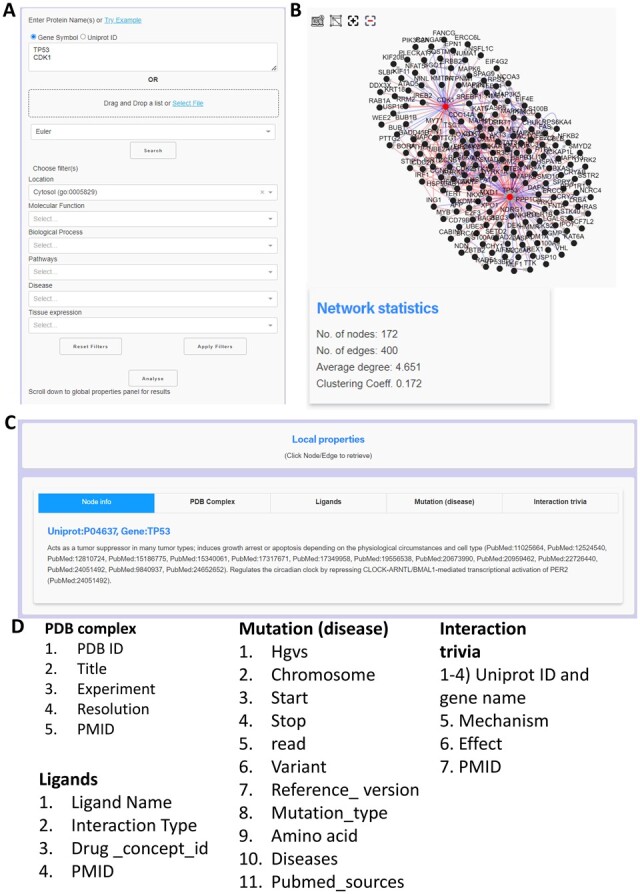
Network construction and the local properties in k2p. (**A**) The directional network is constructed using the query proteins and their first neighbours. To make the network context-specific, we have taken cytosol as an example. (**B**) The constructed directional PPI network. (**C**) The panels where the local properties of the network appear. (**D**) The columns in each local information panel. To better understand these columns, we recommend that readers visit the databases from which this information is curated

**Fig. 4.**
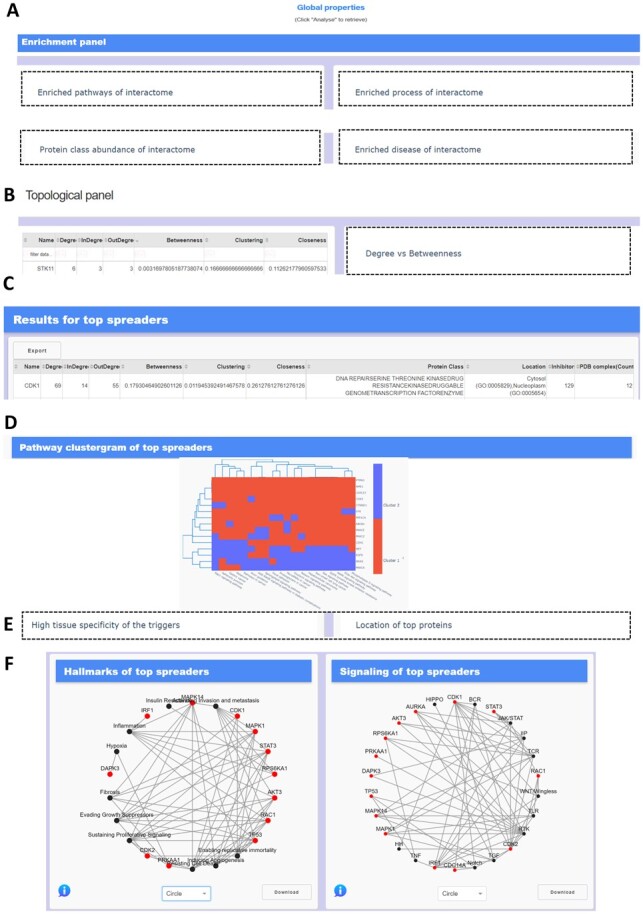
The global properties in k2p. (**A**) The enrichment panels. All the enrichment analysis is done using Enrichr API. (**B**) The topological panel. The topological properties of each node are provided in a tabular format, which can be further sorted in an ascending or descending order. Additionally, a degree-betweenness plot is also provided to find the bottleneck (high-degree high-betweenness) proteins. (**C**) The top 15 IS in the network. Along with their topological properties, k2p also provides the class and structural details of the spreaders. (**D**) The pathway clustergram of the spreaders and associated pathways. (**E**) High tissue specificity and the subcellular localization information of the spreaders. (**F**) Finally, the spreaders are associated with disease hallmarks and the signalling pathways

The network panel is followed by five panels devoted to exploring local properties. These panels offer information about the protein name, structure, ligands, mode of protein interaction and disease mutations. The next pallets contain the enriched pathways, processes, multi-disease interactome and protein class abundance information. The topological properties of each protein, such as degree, betweenness etc., can be found next in the topological panel. The top 15 spreaders in the network can be exported from the ‘top-spreaders’ panel. To further explore the impact of the spreaders, k2p analyses and provides gene-pathway clustergram of these spreaders along with their association with various disease hallmarks and crucial signalling pathways. A snapshot of this web interface can be found in Figures 3 and 4. All the information of k2p can be exported either in a tabular (.xlsx) format or in a graphical (.png and .jpeg) format. The interface of k2p is shown in Figures 3 and 4.

### 3.2 Statistics

**Fig. 5.**
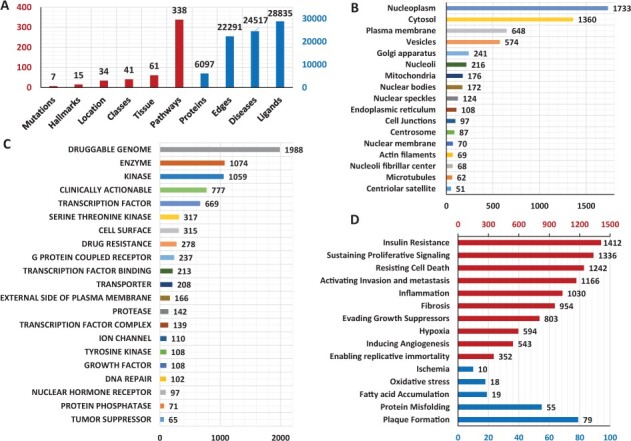
The figure shows the statistics of k2p. (**A**) Different attributes of k2p. The dark red colour represents the primary axis while the blue colour represents the secondary axis. (**B**) The numbers of proteins in each subcellular localization. The subcellular localizations containing <50 proteins are not shown in the figure. (**C**) Classes of proteins. The classes containing <50 proteins are not shown in the figure. (**D**) The distribution of proteins across different disease hallmarks in k2p. The dark red colour represents the primary axis while the blue represents the secondary axis

In the current version, k2p has 6097 proteins with 22 291 interactions between them. It contains information about 24 517 diseases and syndromes, 338 pathways, 34 Subcellular localizations, 42 classes, 61 tissues, 28 835 ligands, 7 different types of disease mutations and 15 disease hallmarks. The information of ligands is available for 2267 proteins, disease and conditions information is available for 4462 proteins, the protein class information is available for 3464 proteins, the 7 types of mutation cover 121 proteins while the 4522 proteins of k2p are scattered across 338 pathways. The structural information for all these proteins is available in k2p. All the statistics of k2p are shown in Figure 5.

### 3.3 Hand-in-hand walk with k2p: a glimpse of the features

The primary objective of k2p is to build a web application that could identify a set of IS from a directional network with a specified context. The interpretation of the spreaders can vary according to the context of the study. For example, in a network formed using differentially expressed genes (DEGs) associated with a disease, the spreaders could be interpreted as triggers, whereas, in a therapeutic network (constructed using DEGs associated with a drug/medicine), the spreaders could be interpreted as the targets. The former is responsible for the disease’s development, whereas the latter may pave the path for a possible recovery. The application of k2p depends on the user queries and interpretation perspectives. However, for a better understanding of the strength of k2p, we are presenting two examples showing the application of k2p in identifying triggers and targets respectively.

#### 3.3. 1 Trigger identification

**Fig. 6.**
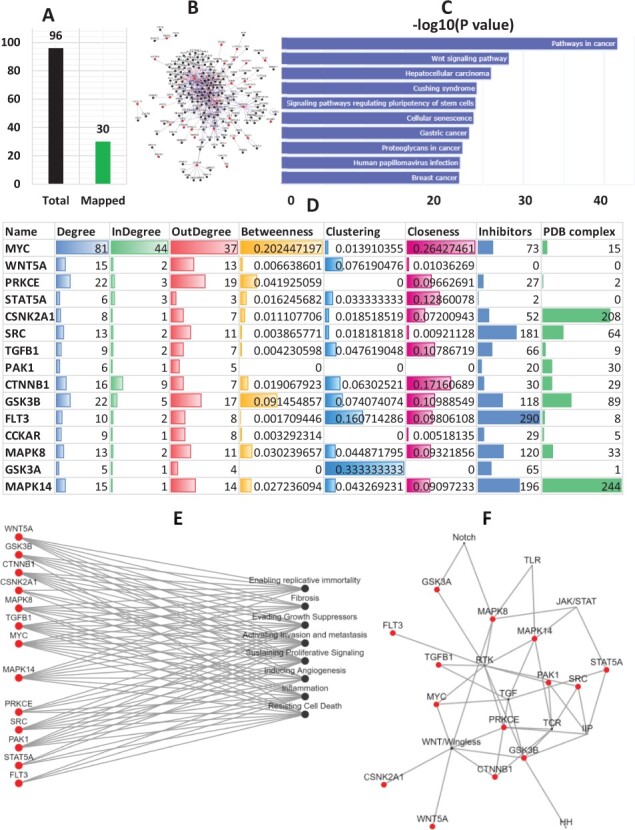
Identification of triggers using k2p. (**A**) The numbers of DEGs mapped to k2p. (**B**) The directional network of DEGs and their first neighbours. (**C**) The KEGG pathway enrichment analysis of the network proteins. (**D**) The top15 IS. (**E**) The association of spreaders with disease-related hallmarks. (**F**) Association of spreaders with crucial signalling pathways

For the identification of triggers using k2p, we took NAFLD as a case study. To identify some potential triggers of NAFLD, we applied k2p to the set of DEGs from the study done by Jia and Zhai (2019). As the literature cramps in terms of the number of directional interactions between proteins, we found most DEGs are not present in the k2p knowledge base (Fig. 6A). The PPI network of the mapped DEGs and their first neighbours are shown in Figure 6B. The enrichment analysis revealed that the proteins in this network are enriched in the WNT-signalling pathway (Fig. 6C), an important target for treating NAFLD. Topological analysis of the network has revealed that MYC is the high-degree high-betweenness gene in the network. It was also the top spreader in the network, followed by WNT5A, PRKCE, STAT5A and CSNK2A (Fig. 6D). The pathway clustergram of these spreaders has revealed that these proteins are sharing multiple pathways. This may lead to the notion that these spreaders are inherently associated with each other, there exist functional relationships between them, and they are functioned to do similar tasks. The protein-hallmark bipartite network has revealed that these proteins are associated with NAFLD hallmarks, such as fibrosis, angiogenesis, inflammation etc. (Fig. 6E). The association with evading growth suppressors has indicated that these proteins may be responsible for advancing the disease towards the cirrhosis stage of NAFLD. The pathway–protein bipartite network has revealed that these proteins are associated with pathways like WNT-Signalling, hedgehog, NOTCH, TGF etc. (Fig. 6F), which are strongly associated with the development and progression of NAFLD. Summarizing all, these top-spreaders are (i) topologically significant in the DEG network, (ii) capable of efficient spreading of information throughout the network, (iii) associated with crucial NAFLD related hallmarks and pathways, and therefore, can trigger the progression of the disease following their inhibition or overexpression.

#### 3.3. 2 Target identification

**Fig. 7.**
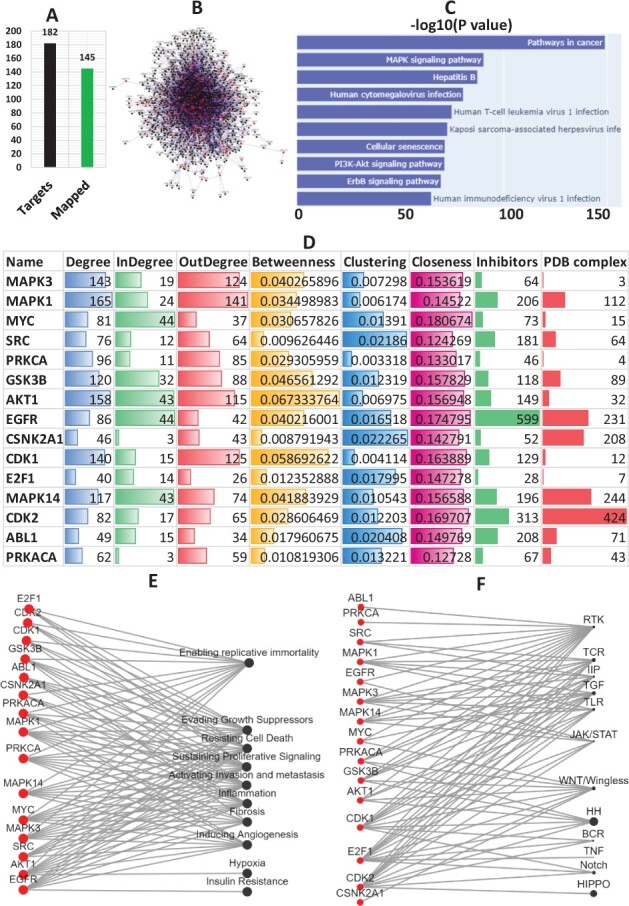
Identification of targets using k2p. (**A**) The numbers of XHTs mapped to k2p. (**B**) The directional network of XHTs and their first neighbours. (**C**) The KEGG pathway enrichment analysis of the network proteins. (**D**) The top15 IS. (**E**) The association of spreaders with disease-related hallmarks. (**F**) Association of spreaders with crucial signalling pathways

To identify targets using k2p, we took Xihuang pill (XH) as a case study. It has been used to treat breast cancer in traditional Chinese medicine (Yang *et al.*, 2020). We began by extracting XH targets (hereinafter referred to as XHT) from Wu *et al.* (2020) and mapped them to the k2p knowledge base. Figure 7A illustrates the number of mapped XHTs. The directional network of XHTs and their first neighbours are shown in Figure 7B. The top 10 enriched pathways of these proteins include the cancer pathway, MAPK signalling pathway, PI3K-Akt signalling pathway etc (Fig. 7C). Numerous drugs are being tested in clinical studies that target these pathways (Miricescu *et al.*, 2020). Basic topological analysis of the network has revealed that AKT1 is the protein with the highest betweenness while MAPK1 was the hub node in the network. The IS of the XHP interactome are shown in Figure 7D. The mitogen-activated protein kinases, MAPK3 and MAPK1 were found to be top 2 IS, followed by MYC, SRC and PRKCA. The association of the IS with the disease hallmarks and crucial signalling pathways are shown in Figure 7E and F, respectively. Breast cancer is highly progressive and currently, there is a lacuna of established drug targets. Although XHP is traditionally being used as a medicine for breast cancer, its specific mechanism of action is still unclear. We have used the directional interactome constructed using k2p to get the most probable and suitable candidate for targeting, among the plethora of targets of XHP. k2p identified 15 IS, which are associated with crucial biological pathways, and are druggable. Among the identified targets, five proteins, SRC, AKT1, EGFR, CDK1 and ABL1, were already reported as successful targets in breast cancer, suggesting that k2p could be used to identify potential drug targets against a disease.

## 4 Conclusion

The motive of our work was to pull together a large amount of ontological knowledge, parse it down into a single interconnecting functional network, and gain insight into the molecular underpinnings of the disease development by creating a one-stop junction for PPI data. Currently, k2p contains information on 6097 proteins with 22 291 interactions between them. However, the application of certain filters may drastically limit the network size, which will have a substantial effect on the network-based analysis. Therefore, we urge the users to complete this step with extreme care. In the future releases of k2p, we intend to increase these numbers. We will also enhance the analysis part by providing other centrality measures and structural insights in the future releases of k2p. The pipeline will be expanded for advanced users, where they could upload their own transcriptomic/proteomic data, which would be analysed by the k2p to identify triggers/targets.

In summary, we have developed a web application of protein information, which along with pathway, structure, disease mutation and ontological information, also identifies the spreaders of the user given PPI network. The identified spreaders are enriched across the pathways, biological processes, molecular functions and tissue-specific. Moreover, to reduce any false-positive identification, or, in other words, to remove any implausible proteins, k2p connects the identified spreaders to disease hallmarks. Hence, k2p overcomes the customary multi-hopping of diversified platforms by being a one-stop junction, and thus it is safe to assume that it will have an ineffable contribution to the drug discovery and development pipeline.

## Data Availability

konnect2prot is freely accessible using the link https://konnect2prot.thsti.in. The code repository is https://github.com/samrat-lab/k2p_bioinfo-2022.
